# Health-Related Motor Testing of Children in Primary School: A Systematic Review of Criterion-Referenced Standards

**DOI:** 10.3390/children8111046

**Published:** 2021-11-12

**Authors:** Paulina Köster, Andreas Hohmann, Claudia Niessner, Maximilian Siener

**Affiliations:** 1 Institute of Sport Science, University of Bayreuth, Universitätsstraße 30, 95447 Bayreuth, Germany; Andreas.Hohmann@uni-bayreuth.de (A.H.); Maximilian.Siener@uni-bayreuth.de (M.S.); 2 Institute of Sports and Sports Science, Karlsruhe Institute of Technology, Engler-Bunte-Ring 15, 76131 Karlsruhe, Germany; Claudia.Niessner@kit.edu

**Keywords:** motor skills, children, health, cut-off points, motor test, fitness assessment, ROC curves, criterion-referenced, physical fitness

## Abstract

Being physically fit in younger years prevents several diseases in the presence as well as in the life course. Therefore, monitoring physical fitness and motor competence through motor testing is essential for determining developmental status and identifying health-related risks. The main objectives of this systematic review were (1) to identify currently available health-related criterion-referenced standards and cut-off points for physical fitness and motor competence test items, (2) to frame the methodological background on setting health-related criterion-referenced standards and (3) to give implications for a health-related evaluation system for physical fitness and motor competence tests. The electronic data base search (PubMed, Web of Science and SURF) yielded 2062 records in total and identified six empirical studies reporting cut-off points of motor test items for children (7–10 years), as well as 30 methodological papers discussing determination approaches to health-related criterion-referenced standards. Data collection, selection and analyses followed the PRISMA guidelines. Health-related motor test standards need to be gender- and age-specific but should refer to an absolute cut-off point rather than to relative performance in the reference group. Due to the lack of data on health-related criterion referenced standards, receiver-operating-characteristic (ROC) curves provide a tool for the determination of cut-off points and criterion referenced standards for physical fitness and motor competence tests. A standardized approach forms the fundamental base for a globally applicable evaluation of health-related fitness tests.

## 1. Introduction

Insufficient physical activity (PA) together with lack of physical fitness (PF) and motor competence (MC) seem to be the new epidemic of the 21st century. PF is represented by the overall performance in various strength, speed, and endurance tasks in a specified physical, social, and psychological environment [1]. Tests for the assessment of PF are a substantial part of many general test batteries, such as the “German Motor Test 6–18” (GMT) [2]. Motor competence (MC) summarizes the degree of proficiency in a wide range of motor tasks, as well as the movement quality, coordination, and control leading to a particular motor performance [3,4]. PF as well as MC are key factors of PA, of well-being, and of physical, psychological, and social health in short and long-term development [5,6,7]. Accordingly, Stodden et al. [7] identified MC and the mediators PF and perceived MC in their conceptual model as key factors enhancing or lowering the PA levels of children, which directly influences the risk of obesity and healthy development of children.

Although the multifaceted phenomenon of PF and MC in younger years is known as a positive marker of health in both the presence and the life course [8,9], recommendations of the World Health Organization (WHO) on PA, PF, and MC for children [10] have received only little attention in recent years. As a result, the increasing and predicted numbers of obesity, decreasing PA, deficient PF and MC in children are connected with several health risks and have triggered concerns in public discussions [11,12,13]. Further, deficient PA, PF and MC are considered contributors to the increasing number of obese children and adolescents [14]. In turn, the positive impact of PF and MC on health in children and youth is attached with rising importance and is being intensively treated in pediatrics [8,15]. Monitoring PF and MC through motor testing becomes essential for determining developmental status, identifying health-related risks, developing recommendations based on motor test results and achieving specific levels of PF and MC [14,16,17,18,19,20,21].

Motor testing can be performed using either product- or process-oriented assessment tools. Whereas the PF testing is dominated by the product-oriented approach focusing solely on the quantitative outcome of the skill execution, in MC assessment also process-oriented tests are used judging the quality of behavioral criteria of the skill execution. From the only moderate correlations between the results of product- and process-oriented assessments of MC, it was concluded that the two approaches measure somewhat different constructs of MC [22]. So, in the following we focus on the evaluation of product-oriented motor test results, which comprises the interpretation of achieved test scores and represents the most relevant part for a meaningful use of the results in health promotion of children. The focus on product-oriented test results is also justified by the fact that there is substantial overlap, in content between motor competence and physical fitness assessments **[23]**, culminating in a one-dimensional structure for 6- to 9-year-old children found by Utesch et al. [24] within the item response theory (partial credit model) for the GMT (excluding the flexibility test).

With the start of youth fitness testing more than 60 years ago, the evaluation process was characterized by a performance-related perspective. This focus arose from the pursuit of military preparedness during times of war and also from the general growth of interest in sports in schools and the society. Recognizing the linkage between fitness and military preparedness along with a very low fitness status in American children led to the development of the fitness youth test AAPHER (American Alliance for Health, Physical Education and Recreation) that was first published in 1958 [25,26]. In Europe, the awareness and efforts for monitoring fitness of children and youth occurred 20 years later than in America. In 1978 the fundament for the EUROFIT test battery was set. It was finalized and presented as the EUROFIT handbook in English and French in 1988, integrating both motor performance measures (strength, power, speed, flexibility, balance, endurance) and body composition measures (height, weight, and body fat). The responsible initiative of the Council of Europe, the Committee for the Development of Sport (CDDS), strived for the development of population-based references in all European countries [27]. 

The health-related perspective on fitness test evaluation arose out of various factors and incidents. The end of the cold war, higher awareness, and knowledge of the influence of PA, PF and MC on health [4,5,7,28] as well as the evolution of exercise physiology and measurement, among other factors, have triggered the transition from performance-related to health-related fitness perspective. Due to the enhanced understanding of the connection of low levels of PF and MC with obesity and risk of chronic diseases, interest in health-related fitness testing continued to grow in the 1980s [29,30]. The higher awareness for health led to a shift towards health-related fitness testing, which has been associated with increased relevance for criterion-referenced evaluation frameworks [31]. 

Basically, there exist two main evaluation frameworks in product-oriented motor testing, which assesses the outcome of a movement: the norm-referenced and the criterion-referenced approach ((pp. 129–153 [32]), [33]). One approach should not be considered the better one, but for the evaluation it is important to choose the appropriate method in the respective context. Norm-referenced tests assess the performance level of an individual in relation to the performance in the reference group. The corresponding norm-referenced standards are based on age- and gender specific population distributions and are characterized by a relative classification accordingly. Using the collected data, percentile ranks or category ranks, e.g., based on z-score classifications, are determined, and classify an individual’s result compared to the results in the reference group (e.g., age- and gender-specific). Thus, norm-referenced evaluation is valuable for inter- and intrapopulation comparisons and in performance-linked settings ([19], (pp. 129–153 [32]), [33,34]). To date, norm-referenced standards are also mainly used for the interpretation of motor test data in children [35,36,37]. Data sets of different motor tests, such as the German “Motorik-Modul” (MoMo) [32], the GMT 6–18 [2], the Motor Competence Assessment [38] or the “Körperkoordinationstest” (KTK) [39] provide representative percentile curves and evaluate the state and development of motor competence in children compared to their reference group ([15], (pp. 129–153 [32]), [35,36,40,41,42]). In particular, Luz et al. [20] highlight the stable positive influence of MC on health-related fitness in childhood and early adolescence.

In comparison, criterion-referenced standards (CRS) classify results in relation to a certain field of skills. The test score is checked against a predetermined absolute criterion or cut-off point without dependence on the reference group and categorizes participants in meeting or not-meeting the CRS afterwards [31,33,43,44]. However, the terms “standard” and “cut-off point” are often erroneously used synonymously, even though they are more of an inferential relationship. Kane’s [45] definition supports a better understanding and differentiation of these two terms:

*“The performance standards provide qualitative descriptions of the intended distinctions between adjacent levels of performance (e.g., between acceptable performance and unacceptable performance). The cutscores are points on the score scale, with one cutscore associated with each performance standard. The cutscore provides an operation version of the corresponding performance standards.”* (p. 55)

Accordingly, the CRS describes an abstract, though observable performance which is operationalized by an absolute cut-off point representing a minimum score for achieving the CRS [46]. In order to obtain valid CRS for health-related fitness, a clear association between the standard and the health indicator needs to be recognized [33,47]. Taking this into account, the criterion-referenced evaluation framework represents an approach that allows to define motor test cut-off points and CRS in relation to specific health criteria [19,26,27,29,30,33]. In this context, health criteria are defined as measurable indicators or risk factors that have been shown to be associated with disease, e.g., high BMI with obesity or high blood pressure with cardiovascular disease. Health-related criterion-referenced evaluation supports the diagnostic of PF and MC as well as the planning of intervention programs. In comparison, the norm-referenced approach is a proven tool in performance-oriented domains due to its relative evaluation in specific populations [33].

There are various test profiles used for assessing PF and MC [2,32,38,39]. In Germany, the generally recognized GMT [2] is probably the best-known test profile and its test items are partially or even completely represented in the national test campaigns [2,32,37,48]. These test variables of the GMT cover the PF domains of endurance, strength, and speed, as well as the MC domains of gross motor skills and fundamental movement skills, like basic stability (e.g., balance), locomotor movements involving two or more body segments, (e.g., jumping), motor coordination and flexibility [23]. Based on the eight tests of the GMT (20-m sprint, backward balancing, 15-s sideward jumping, standing long jump, 40-s push-ups, 40-s sit-ups, standing bend forward, 6-min endurance run) Hohmann et al. [37] developed the Fulda Movement Check (FMC), a test battery consisting of a total of eleven test items. Due to the absence of the assessment of maximum strength, agility, and object control in the tests of the GMT, the FMC consists of a handgrip strength test, a 10 × 2-m change of direction agility test and an 80-gr ball throw test and thus covers a wide range of PF and MC components. The international-known Motor Competence Assessment, which tests sideward jumping, standing long jump and ball throwing, among other tests, and KTK, which also includes balancing backwards and sideward jumping, are largely consistent with the FMC test battery. However, these tests use an evaluation system that assesses a child’s MC by calculating sub-scales of stability, locomotor, and manipulative skills (Motor Competence Assessment), or total scores in percent % (KTK). Therefore, the generalized test items of the FMC are taken as the basis for the analysis of CRS in this study. 

Contradictory to the health-related approach, children’s test results have been assessed on the basis of norm-referenced approaches so far. To provide an evaluation framework that is able to differentiate between children with and without health risks, health-related CRS are required for this test battery. The respective cut-off point indicates a minimum level of PF and MC that is needed to live free of disease or even gain health benefits. The concerning increase of obesity and decrease of PF and MC in children in combination with the small body of research on health-related CRS for field-based motor tests, reveal the high relevance of health-related CRS. 

The aim of this systematic review was to identify empirical studies reporting health-related CRS for FMC motor tests in children aged seven to ten years (primary school) as well as methodological papers discussing approaches to the determination of health-related CRS.

## 2. Methods

### 2.1. Data Sources 

Data collection, selection and analyses followed the PRISMA (Preferred Reporting Items for Systematic review and Meta-Analysis) guidelines [49]. The study search was based on the following electronic data bases: PubMed, Web of Science and SURF. The results of the data base searches revealed a total of records in the time period from January 1972 up until April 2021. Accordingly, the oldest article found in the review research dates back to 1972. The search strategy was built out of search term modifiers that covered four general sets: (1) motor skills, (2) assessment, (3) standard and (4) children. The study search was limited by the languages of English and German. Appendix A includes the full search strategy conducted in Pubmed. This review protocol was registered on the International Prospective Register of Systematic Reviews (PROSPERO; registration number: CRD42021285860).

### 2.2. Inclusion Criteria 

The systematic review includes scientific articles that determine health-related CRS or rather cut-off points for at least one of the FMC motor test items on the one hand, and methodological papers evaluating approaches to the determination of health-related CRS for motor tests on children on the other hand. Using the PICOS (population, intervention, comparison, outcome and study type) framework [50] as an orientation, the eligibility criteria for empirical studies were defined as follows: (1) apparently healthy children (free from disease or injury) from seven to ten years old, (2) field-based motor test including at least one of the following FMC items: 20 m-sprint, standing long jump, stand-and-reach, ball throwing, jumping sideways, push-ups, sit-ups, balancing backwards, change of direction agility, 6-min run and handgrip strength test, and (3) determination of health-related cut-off points or CRS. Methodological articles were included if they addressed the setting process of health-related CRS for motor tests in children.

### 2.3. Study Selection

All located references were imported into a reference management software (Zotero 5.0.96.3, Corporation for Digital Scholarship, George Mason University, Fairfax, VA, USA). Duplicated studies were automatically removed by Zotero, the remaining duplicates by manual removal. One reviewer (P.K.) screened titles and abstracts of the remaining references for potential relevance. Full-text copies were attached to all studies that fulfilled the initial screening criteria. Afterwards, the selected full-text articles were screened against the eligibility criteria and a reference list with all included articles was compiled. Additional studies that were identified by screening the reference lists of relevant studies were added to the results of the electronic search.

**Figure 1:**
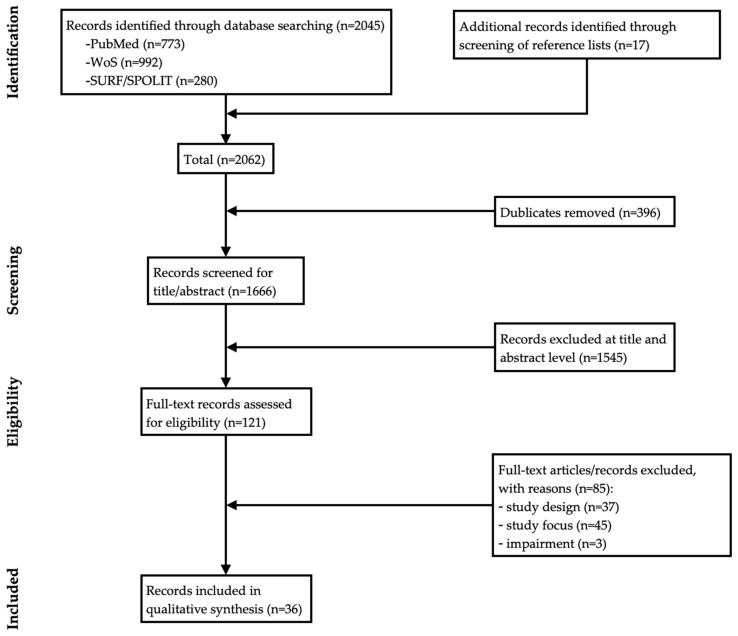
PRISMA [49] flow diagram illustrating the systematic search and review process.

The electronic data search yielded a total of 2045 records that met the search strategy criteria. These records were complemented by 17 additional records identified by screening reference lists. After removing duplicates, 1666 records were included in title/abstract screening. An initial screening revealed 121 records that were considered potentially relevant. The full-text reading process excluded 85 records with reasons resulting in 36 article (6 empirical, 30 methodological) that were considered eligible for inclusion. Figure 1 shows a PRISMA [49] flow diagram illustrating the details of the selection process of studies with reasons for exclusion. 

### 2.4. Data Collection and Analysis

Data-extraction forms were created by the first author and were reviewed by a second author (M.S.). For the empirical part the characteristics of the study (author, year of publication, sample size and age), the characteristics of the motor test and health indicator, and information on determined gender-specific cut-points were extracted. For the methodological part the characteristics of the study (author and year of publication), the methodological context for this systematic review and the main content of the article were extracted. During data extraction, reviewers were not blinded to the authors or journals. Based on the reported exposure and the grouped studies, the results are presented as a narrative synthesis.

## 3. Results

### 3.1. Health-Related Criterion-Referenced Standards for Motor Test Items of the Fulda Movement Check

The FITNESSGRAM© youth fitness program was introduced in 1982 as the first “student fitness report card” and integrates health-related CRS since 1987. Today, it enables better planning of physical education in schools by supporting the collection and processing of data on student’s PF and PA [30,51]. Originally, a single cut-off point for each Fitnessgram test item embodied the minimum performance level needed for health or minimal disease risk, independent of the performance in the reference population [30]. The process of setting cut-off points involved empirical data, normative data and professional judgment of an advisory council [52]. In 1992, the “Healthy Fitness Zone” concept was introduced and replaced the original concept of single cut-off points. Since then, the test results are classified based on the “Needs Improvement Zone”, the “Healthy Fitness Zone” and the zone above the healthy fitness zone. In 2011, the healthy fitness zone cut-off points for body composition and cardiovascular fitness tests were updated based on the link between metabolic syndrome risk and fitness test results [53]. Receiver-operating-characteristic (ROC) analysis, a method for setting cut-off points which is described in detail in Section 3.3, was used to define the optimal cut-off point distinguishing between healthy children and children with higher risk for metabolic syndrome disease [30]. Table 1 includes the age- and gender-related cut-off points for the push-up test and the curl-up test as a variation of the sit-up test. However, these CRS are not determined on the basis of health outcomes, but on training response to exercise. The authors reasoned this methodology based on the delayed effects of poor musculoskeletal fitness on health risks in children [54]. 

Castro-Piñero et al. [55] identified muscle fitness cut-off points that distinguish between low and high risk of cardiovascular disease in children (6–10 years) and adolescents (12–16 years) via ROC analysis (Table 1). Muscle fitness was measured by handgrip strength and standing long jump, whereas the cardiovascular disease risk score was composed of the z-scores of two skinfolds, systolic blood pressure, insulin, glucose, triglycerides, and total cholesterol/high density lipoprotein cholesterol. The defined cut-off points were still true for the detection of cardiovascular disease risk in a longitudinal follow-up after two years. Moreover, the associations between muscle fitness and cardiovascular disease in the cross-sectional and the longitudinal approach are not influenced by cardiorespiratory fitness strengthening muscle fitness as a health marker in these age groups. 

Besides, Castro-Piñero et al. [56] determined muscle strength cut-off points associated with risk of metabolic syndrome in European adolescents. Upper and lower muscle strength were assessed by handgrip strength and standing long jump respectively. By measuring of waist circumference, diastolic and systolic blood pressure, triglycerides, high- density lipoprotein cholesterol and fasting glucose, metabolic syndrome status was identified using a specific classification proposed by Jolliffe and Janssens [57]. In addition, a continuous cardiometabolic risk index based on four cardiometabolic risk factors (waist circumference, mean arterial pressure, triglyceride/high density lipoprotein ratio and fasting insulin) was calculated that gives further insight into the metabolic health of participating adolescents. Finally, age- and sex-specific cut-off points for absolute and relative handgrip strength and standing long jump were determined in relation to metabolic syndrome and the continuous cardiometabolic risk index using ROC curves. Regardless of age, which ranged from 12.5 to 17.5 years in participants from nine European countries, this study still serves as an example for setting cut-off points for the handgrip strength and standing long jump test indicating cardiometabolic risk independent of cardiorespiratory fitness. Due to the disagreement in age with the inclusion criteria, these cut-off points are not included in Table 1.

In contrast to Castro-Piñero et al. [56], limited validity of handgrip strength as a screening tool for cardiometabolic risk in children was reported by Fredriksen et al. [58]. In the Health Oriented Pedagogical Project, the relation of cardiometabolic risk and handgrip strength in six to 12-year-old children was examined. High values of systolic blood pressure, waist circumference, total serum cholesterol and percent body fat are stated as cardiometabolic risk factors. Consequently, the sum of z-scores of these values were used in this study as a measure of total cardiometabolic risk. Based on ROC analysis applied to BMI, handgrip strength, waist circumference and waist-to-height-ratio, the variable of BMI was identified as the most appropriate cardiometabolic risk factor. The predictive quality of handgrip strength to display cardiometabolic risk was significantly lower compared to the other measurements. Accordingly, these results disagree with the findings of Castro-Piñero et al. [56] and do not indicate handgrip strength as a screening tool for cardiometabolic risk (see Table 1).

Saint-Maurice et al. [59] examined the association between handgrip strength and bone health in children and youth. Handgrip strength measured by handheld dynamometer represents a proxy measure for full body strength and therefore serves as an indicator for bone health [60]. Using data from the Iowa Bone Development Study (IBDS), a purposive sample of healthy youth from Midwest USA (1998–2004) in combination with a random sample of healthy boys and girls from Spain in the Healthy Lifestyle in Europe by Nutrition in Adolescence (HELENA) (2006–2007), handgrip strength cut-off points in relation to bone health (see Table 1) were established by ROC analysis and cross-validated afterwards. As a result, three health zones of “low risk”, “some risk” and “high risk” were created. The results have shown acceptable classification accuracy for identifying healthy bone development. This study provides insights into setting health-related CRS for handgrip strength using bone health as a health indicator.

Recently, Santos Silva et al. [61] determined performance cut-off points for the 9-min walk/run test in relation to the BMI-classification of Brazilian boys and girls at the age of 6–17 years. Because obesity is known as a well-studied health risk indicator and aerobic fitness has been reported to improve body fat levels of children, the link between BMI and 9-min run/walk performance is of interest. Cut-off points which refer to the minimum performance required for low risk of obesity were also identified using ROC curves. Though, it needs to be considered that the FMC contains the 6-min run test rather than the 9-min run test. Consequently, the values for the 6-min run were calculated using a simple equation formula to approximate the corresponding cut-off points, even though the validity of the results is questionable (see Table 1).

Summarizing the main results of the six empirical studies, it is apparent that ROC analysis is predominant in setting CRS on the one hand, and that there is no agreement yet on a specific health indicator for motor testing. Moreover, it becomes clear, that the data base is limited and will not allow general conclusions on CRS. 

### 3.2. Body Composition as a Health Indicator in Motor Testing

The relation of overweight and obesity or rather higher BMI-categories with inferior performances in motor tests was recognized during the last years [62,63,64,65,66,67,68,69] (see Table 2). For example, Zaqout et al. [65] who examined the effect of PF on cardiometabolic risk factors found a strong correlation between higher waist circumference and both lower limb strength and cardiorespiratory fitness in European children (6–11 years). In addition, the findings of Ruzbarska [66] contain significantly poorer MC in overweight and obese children compared to their normal-weight peers. Further, Tokmakidis et al. [63] examined the relationship between body composition and PF performance in Greek primary schoolchildren and support the limiting influence of overweight and obesity on the PF level. Similarly, a decline in aerobic capacity and muscle strength (standing long jump, knee push-ups, sit-ups, wall sitting and aeroplane lying) was shown with increasing BMI in South African children (9–13 years) by Truter et al. [69]. The negative effects of body composition on physical performance were significant in the aerobic fitness test (PACER) between normal-weight, overweight and obese children, and in the standing long jump and knee push-ups between normal-weight and obese children. As pointed out in Section 3.1, Santos Silva et al. [64] researched the context of body composition and PF in children and youth (6–17 years) from Brazil and identified BMI-referenced cut-off points for the 9-min run test, which is close to the 6-min run of FMC. Thus, the relevance of BMI as a valuable health indicator in PF testing was emphasized. Likewise, Silva et al. [70] found a negative association of cardiorespiratory fitness performance and obesity in 10-year-old Canadian children. However, this was assessed by the non-FMC test of shuttle run. They determined corresponding BMI-, waist circumference- and combined BMI- and waist circumference-referenced cut-off points for cardiorespiratory fitness using ROC analysis with good discriminatory power for obesity. 

Nevertheless, there also exist controversial studies that do not show any difference of PF and MC levels between BMI categories in children. Lovecchio and Zago [71] found similar results in sit-and-reach, standing long jump, shuttle run (10 × 5-m) and sit-ups tests in normal, obese and thin students classified by BMI cut-off points. Ervin et al. [72] even found positive effects of higher BMI classification on muscle strength in tests that do not involve lifting the body.

### 3.3. Approaches to Setting Criterion-Referenced Standards and Cut-Off Points for Health-Related Evaluation

Several different approaches are discussed in the literature for setting CRS in motor testing. A total of 20 articles addressing this topic were included from the study search (see Table 2). 

Cureton and Warren [52] already discussed these setting approaches in 1990 and differentiated four main categories: 1. judgmental, 2. normative, 3. empirical and 4. a combination of 1.–3. Zhu et al. [26] referred to a simplified categorization containing two evaluation frameworks, the test-centered and examinee-centered approach. They are aligned with the first and third approach of Cureton and Warren [52]. It should be noted that the discussed approaches in the following sections do not only work separately, but also in combination, which might prove useful regarding the complexity of CRS.

The judgmental or rather test-centered approach is characterized by a qualitative analysis. It uses a panel of experts who determine a cut-off point of each test item through the application of their expertise. This cut-off point describes the acceptable minimum level of the criterion [26,43]. The “Angoff-Method” is a well-known example of a test-centered approach in which a panel of judges set the expected performance of a minimally competent student on each test item. By assessing the person who is just achieving a performance to pass the test standard, the cut-off point for each test item is determined [46]. One part of the original setting process of Fitnessgram cut-off points contained a judgmental valuation [51]. 

In the second approach, normative data are combined with relevant information to set cut-off points [52]. Different norm-referenced cut-off points based on data, such as percentiles or growth curves, exist that identify low performance associated with health risks [19]. Some authors choose the 10th percentile as the minimum performance required [79], others the lower quintile or the lower quartile [80], while still other authors refer to test results below the 50th percentile as hazardous to health [52,75]. Moreover, several norm-referenced tests use a Likert type scale for classification that differentiates PF and MC, for instance, as follows: very poor (X < P10); poor (P10 ≤ X < P25); medium (P25 ≤ X < P75); good (P75 ≤ X < P90); and very good (X ≥ P95) [75,81]. The MoMo test and the GMT are appropriate examples that provide indications of a child’s health status based on normative data and percentiles ([15], (pp. 129–153 [32]), [34,35,36]). McArthur et al. [82] revised the scaling of the Acquired Brain Injury Challenge Assessment (ABI-CA). Even though this test relates to impaired children, it serves as an example of using normative data to determine cut-off points. By testing typically developing children and calculating mean and standard deviation (SD) of the results, cut-off values were identified which differentiate between healthy and impaired children. Depending on the number of cut-off points required, either one or two standard deviations were added to and subtracted from the mean value respectively. Besides, the norm-referenced approach can also serve as a tool for verifying CRS. Santos et al. [83] used PF percentiles of Portuguese youth to verify the agreement with the Fitnessgram healthy fitness zones. 

The empirical or examinee-centered approach makes use of empirical data. Further, this approach can be divided into the borderline-group and the contrasting-group method [43]. The borderline-group [52] or criterion test technique [73] involves a combination of an external measure of the criterion attribute/behavior with empirical data. Consequently, a cut-off point of the criterion indicated through available data can be directly applied to the performance on a test. Nevertheless, this method is still dependent on some judgements as well as a strong relationship between the test results and the external measure [26,44,52,73]. In comparison, the contrasting groups method compares a master group with a non-master group in a specific test to identify the most appropriate cut-off point. Plowman [73] recommends following four steps in the determination process of CRS using the contrasting groups method: 1. Identifying two discrete groups, one master and one non-master group. 2. Plotting the frequency distribution of both groups. 3. Setting of the cut-off point in the overlap point of the two distributions. 4. Statistical evaluation of the identified cut-off score (correlation between the contrasting groups, validity, utility analysis). 

An extension of the contrasting groups method is the ROC curve method that supports the determination of the “optimal” or rather most appropriate cut-off point by making use of sensitivity and specificity. Sensitivity describes the true-positive rate (test correctly identifies a positive result for examinees who have the tested competence/skill) and specificity describes the true-negative rate (test correctly flags the examinees who do not have the tested competence) [73,74]. Based on post-examination data and a predetermined cut-off score of the health factor the respective score distributions are plotted, and an analysis of sensitivity and specificity determines the cut-off point for the test performance. All possible cut-off points contrasting sensitivity with specificity are reflected in the ROC curve. Consequently, the optimal cut-off point integrates the highest sensitivity with the highest specificity and allows a dichotomous classification of the health state in healthy or with disease risk (in the clinical context) [77,84]. Additionally, the cut-off point can be adjusted depending on the test characteristics by different weighting of sensitivity or specificity. In cancer treatment, for example, great importance is attached to high sensitivity, while lower specificity is accepted [84]. However, there exist different approaches to the determination of the optimal cut-off point, e.g., the Youden index (J) method which identifies the optimal cut-off point by increasing the difference between the true positive rate and the false positive rate to the highest possible level. Moreover, ROC curves also determine the diagnostic accuracy of a biomarker. The area-under-the-curve is a proven measure for this purpose [76]. 

ROC analysis has been used to set cut-off points by contrasting two groups in several studies [55,56,58,59,61,85] in recent years (see Table 1). Lang et al. [78] conducted a review of CRS for cardiorespiratory fitness. A search of four databases identified papers that determined CRS using ROC analysis in children and youth. Though the review refers to the 20-m shuttle-run test as the field-based assessment tool for cardiorespiratory fitness, which is not included in the FMC test battery, it outlines the ROC analysis as a tool for setting CRS in health-related fitness testing. Moreover, the new CRS of the aerobic (VO_2_max) and body composition (body fat and BMI) measures in the Fitnessgram program were also updated using ROC curves [30].

## 4. Discussion

### 4.1. Determination of Criterion-Referenced Standards in Health-Related Motor Testing

The value of motor testing ranges from its ability to draw a picture of the PF and MC level, to detect health-related risks in children, to monitor PF and MC changes over time and to enable engagement in PA. In their conceptual model, Stodden et al. [7] emphasize the importance of MC level and the mediators PF and perceived MC on PA behavior and associated health risks. MC is pointed out as a key mechanism that influences not only higher or lower PA levels, but also obesity of children. However, the evaluation of the performance of a child in motor testing is reliant on valid and reliable test standards. From a practical point of view, standard values are essential in order to classify test results, compare them, give subsequent recommendations and build individual intervention and prevention programs. The results of all test items in a motor test provide an overall profile that should reveal not only strengths and weaknesses in physical abilities and motor skills, but also health risks of an individual child or even an entire group. Considering the importance of adequate PF and MC to health and thus motor testing in children, the aim of this systematic review was to provide an overview of currently available health-related CRS and cut-off points for motor tests and to develop implications for a health-related evaluation approach. Table 1 and Section 3.1 make clear that the current state of research provides little to no data on health-related cut-off points that could have been used for further analysis. 

Regarding the two evaluation frameworks, the norm- and criterion-referenced approach, CRS are crucial for health-related assessment of PF and MC components. Even though the calculation of norm-referenced standards is relatively easy and quick it has to deal with its limitations: The relative classification causes a strong dependency on the criterion behavior or attribute in the reference group. In a health-related perspective, this dependency results in a limited meaning of the classification: In case of a population that is predominantly diseased/unfit or healthy/extremely fit, an over- or underestimation of an individual’s test result occurs. Beside this, reference data, that are not updated regularly (due to cost, time or manpower), do not reflect true norm values because of possible changes in the population over time [33,43,44,47]. Moreover, a discouraging effect of unfit children [86] together with a tendency of mainly awarding those, who are already fit, can occur in the norm-referenced evaluation framework [26,44]. Although norm-referenced standards are regarded critical in the context of health-related fitness testing [31,73], they can be used as a baseline [18] or even as a starting point for CRS [18,87]. The criterion-referenced approach is based on an absolute criterion, such as a specific health outcome, and is therefore independent of the behavior or attributes of the reference group which mitigates the limitations of norm-referenced standards. Additionally, CRS provide the fundamental framework for cross-validation and evaluation in independent samples and with longitudinal study design [26,43,47]. Kemper and Van Mechelen [27] already pointed out the importance of health-related cut-off points in the EUROFIT test battery in 1996 but underlined the difficulty of setting CRS from a strict measurement perspective. Kane [88] stated “There is no gold standard. There is not even a silver standard” (pp. 448–449), implying that there is no external method which is definitive in identifying valid cut-off points. Although the criterion-referenced evaluation framework is less restricted by limitations compared to the norm-referenced approach in the health-related context, it has to deal with the substantial challenge of setting appropriate standards and cut-off points [26,27,29]. In addition to the challenge of setting test standards, the test characteristics validity, reliability, and the estimation of domain scores have strong influence on the value of a motor test [43,89]. These aspects need to be considered at least similarly in the standard setting process of motor tests.

In terms of the various approaches to the determination of CRS for PF and MC, the contrasting groups method and the ROC curve method in particular have been found to be the most sounded. The judgmental approach is severely limited by the subjective evaluation of the judging experts in setting cut-off points [26,52] and should therefore only be used for subsequent assessment or review. The normative approach has to deal with the limitation of the dependency on the reference group, a result of using normative data. It should only function as an indicator for health-related standards, because it is not based on a connection between a health factor and the motor test. Moreover, the requirements of CRS, such as an absolute cut-off point, are not met [19,44,47,52]. Plowman [73] who analyzed CRS for neuromuscular fitness tests questioned the validity of the normative and judgmental method already in 1992. The comparison of the contrasting group method and the Angoff-method by Van Nijlen and Janssen [46] outlines the value of the empirical method and the use of the ROC analysis in setting CRS. Nevertheless, normative standards can still serve as a starting point and indicator for health-related CRS, although they do not correspond to the key criterion-referenced characteristics that require an absolute cut-off point [18,87]. Based on the available evidence and studies, the examinee-centered approach using ROC curve analysis appears to be most appropriate for setting health-related standards. Furthermore, ROC analysis allows to set more than one cut-off point. Consequently, classification zones, such as needs improvement zone and healthy fitness zone in Fitnessgram, are created that support a more valid assessment of children with borderline test results as opposed to dichotomous classification [30]. In addition, Welk et al. [53] pointed out that the use of z-scores derived from the LMS (Lambda-Mu-Sigma) method in ROC analysis provides a significant advantage as it integrates normal growth and maturation into health-related CRS. 

The determination of health-related CRS requires an absolute desirable level of a specific health criterion, reflecting the threshold that differentiates between a positive and negative health outcome. It turned out essential to verify a strong correlation between the health factor or even the sum of several health factors and the respective motor test for valid CRS and cut-off points, meaningful classification, and interpretation and also the development of intervention and prevention programs [26,44,90]. Generalizations about health status, determined by motor tests, should be made with caution [91]. Oliveira and Guedes [92] examined Fitnessgram cut-off points for the detection of metabolic syndrome in Brazilian boys and girls (12–20 years) by using ROC analysis. However, only the cut-off points of the aerobic capacity test were confirmed to be valid for the detection of metabolic syndrome, whereas the remaining tests (back-saver sit and reach, trunk lift, curl-up, and push-up) indicated low accuracy in representing the threshold for cardiometabolic risk in Brazilian adolescents. Similarly, Fredriksen et al. [58], who analyzed handgrip strength in children, could not confirm this motor test as suitable for predicting cardiometabolic risk.

As there are several ways to measure health, it is of great importance to focus on one health field when setting test standards, e.g., orthopedics, cardiovascular or cardiometabolic health, mental health, or body composition, in order to avoid misclassification. Table 1 and Section 3.1 make clear that it does not exist a consistent agreement on a particular health factor that should be used for health-related motor testing. Besides, the literature does not offer an absolutely correct recommendation for a particular health measure either [93]. The most appropriate health factor for the respective context should be selected based on current evidence and knowledge [26]. Many campaigns and also the FMC include BMI and Broca Index to assess body composition as an indicator for health. 

Obesity presents a major risk factor for several diseases, such as metabolic syndrome, high blood pressure, atherosclerosis, heart disease, diabetes, high blood cholesterol, cancers and sleep disorders [94]. An early treatment of obesity in childhood is important to reduce premature mortality and physical morbidity in adulthood [95]. BMI and Broca Index both calculated with body weight and height are used as biomarkers for obesity or rather body constitution and allow indications on the anthropometric health. Nevertheless, the validity of these indices is also weakened by some limitations. Since only the total weight is included, misinterpretations of persons with the same weight, but different fat and muscle mass may occur. Still, it has been shown that the BMI and Broca Index serve as health risk factors in detecting overweight [94,95]. Further, a multifaceted evaluation of a child’s physical health requires motor testing, e.g., by FMC, to assess PF and MC components. The assessment of PF and MC supplemented with body composition evaluation provide a multifaceted picture of a child’s health and development state and of one child. High validity of test standards requires detailed research and evidence regarding the relationship between PF, MC, and health determinants. In this case, the negative impact of obesity on the results of the selected tests needs to be examined. Several studies have shown negative effects of overweight and obesity on PF and MC [62,63,66,67,96] and suggest these factors as appropriate health indicators for motor testing accordingly. However, the associations of body composition parameters with PF and MC were not clear in all studies [71,72]. Some authors have even been able to demonstrate positive effects of increasing BMI on muscle strength [72] and the evidence of a negative correlation of flexibility and handgrip strength with obesity has not yet been proven [47,90,97]. Contradictory studies regarding the relationship between body composition, physical abilities and motor skills need to be considered and imply the need of further research in this field [90]. In addition, gender differences in weight adaptations to physical training and exercise have been observed, which need to be especially considered in long-term interventions [98]. Moreover, body composition represents only one of multiple health components that can be associated with motor testing, as shown in Section 3.1. Thus, research in other health fields may indicate divergent standards compared with studies focused on body composition. 

Consequently, a health-related evaluation concept referring to the selected test profile implies in the first step the determination of CRS based on a health criterium or outcome. The BMI-classification as well as the Broca Index as indices for body composition have been established in the field of obesity prevention and PF promotion. Moreover, these indicators are easy to calculate with only relatively low effort in a field test setting. Nevertheless, the contradictory findings of the relationship of body composition and PF and MC require further research. Proving and confirming a significant correlation between the health measure and the motor test item is crucial for reliable and valid evaluation of PF and MC. This linkage should also be studied in longitudinal studies to track development and possible changes at different ages of life [90].

### 4.2. Pedagogical Factors

Handling and processing of children’s motor test results with the aim of valuable health interventions requires a structured, target group-oriented implementation of health-related standards in motor test settings, in addition to a standardized determination process of health-related standards. First of all, the differences in the attitude towards fitness assessments between children and adults need to receive consideration. In comparison to adults, children are not integrated in the decision power whether to take part in fitness testing or how to use the results for further interpretation. In the context of physical education, fitness tests and the use of the results are rather predefined for school children. Hence, the commitment to and motivation for PA in children is not fundamentally given. In turn, promotion of increased PA with higher motivation in children is a relevant goal of motor testing [99]. In order to take the special characteristics of children into account, it is important to consider not only structural approaches but also pedagogical aspects in the process of setting standards.

Already in 1992, Updyke [100] stated the need of CRS in motor testing of children. He based this statement on the importance of test standards that are not depending on the population to prevent frustration of children regarding PA, which is also supported by Silverman et al. in 2010 [99]. Taking this into account, both the test design as well as the evaluation process need to have a high degree of applicability and comprehensibility for children. Moreover, a high level of understanding of the test standards by all stakeholders is essential in a test setting. Otherwise, the understanding of the relevance of the entire test may be compromised. Building a solid understanding and promoting motivation for PF and MC in children can further improve self-esteem, which is regarded a primary benefit and goal of fitness testing [86,100]. Naughton et al. [101] further underlined the high relevance of the link between fitness testing and encouraging children for PA. Significant positive impact of fitness testing on children’s PA behaviors can counteract childhood overweight and obesity by increasing motivation for PA and an active lifestyle.

Wiersma and Sherman [86] have developed instructional strategies for fitness testing that promote motivation, engagement, and enjoyment in children, thereby preventing adverse reactions to fitness testing. The authors underlined again the demotivating effect of normative rankings, e.g., percentile ranks, on especially the intrinsic motivation and clearly recommend CRS. The use of CRS not only has a positive effect on psychological factors. Even controllable factors, such as effort or attention, can also be better focused, while negative influences due to environmental or genetic factors can be reduced [86,99].

### 4.3. Holistic Perspective on Motor Testing in Children

Since several different approaches to motor test structures exist, it is essential to differentiate and precisely define the measurement properties before initiating the standard setting process. The multifaceted motor characteristics of children require a test that takes as many aspects of PF and MC as possible into account to draw a reliable picture of a child’s motor state. True et al. [102] underlined the relevance of understanding the manifold factors that influence the development of a child at given points. Process-oriented measures describe the qualitative dimension of a movement (e.g., movement pattern), whereas product-oriented measures identify motor performance through quantitative dimensions (e.g., speed, number of repetitions, time). Due to different information processing in boys and girls during PF and MC assessments, both the process- and the product-oriented approach should be considered in order to cover as many demands in children as possible. Gender-dependent information processing behavior requires further research for general statements and increased attention in the development of health-related motor tests and its standards. The MOBAK (basic motor competencies) [103] approach represents an example for a motor test that includes test standard structures based on functional features. Functional in this context means that the tests include movement tasks that are required to perform daily activities. Therefore, the test aims to complement traditional motor testing in physical education (PE) with basic motor competencies relating to functional mastery of motor requirements and tasks (e.g., target throwing). In contrast to the functional-oriented MOBAK test batteries, the FMC includes product-oriented tests that quantify the PF and MC level of children and are therefore suitable for statistical analysis. Different emphases in motor test standards, such as result-oriented/functional, process-oriented/qualitative and product-oriented/quantitative assessment, support the value of a holistic approach that enables a multifaceted and most reliable assessment of a child’s physical abilities and motor skills and prevents misinterpretation. 

According to gender-specific information processing behavior, it seems reasonable to include at least product-oriented and process-oriented test standards in field-based test profiles. One possible approach would be to include predefined movement patterns in the test standards in addition to absolute quantitative cut-off points. The process-oriented standard could then be understood as a supplement to or review of the metric CRS, especially for results that cannot be clearly classified in the total rating. 

Due to the lack of research on health-related CRS for motor tests, as discussed in this article, it is necessary to think further outside the box. The Hungarian National Student Fitness Test [104] was developed by the Hungarian School Sport Federation in collaboration with the Cooper Institute. With the aim of integrating health-related criterion-referenced fitness standards into the educational curriculum in Hungary, a software-supported test battery was developed. As the implementation of health-related fitness testing is still in progress in many countries, this partnership should serve as a model for other countries. Obviously, collaboration, cooperation and coordination are more targeted in this context than competitive thinking [29]. 

### 4.4. Implications for Setting Health-Related Criterion-Referenced Standards

To sum up, the following aspects need to be considered in the development of health-related standards for motor tests in children. Firstly, the required performance level associated with health benefits needs to be delineated by CRS. The classification should be gender- and age-specific, but must refer to an absolute level of performance based on health outcomes rather than to relative performance of the reference group. Consequently, comparability between different motor tests and different times of testing is provided. Due to their link between motor test and health factor, CRS not only include higher informative value about health status, but also prevent frustration and even promote motivation for PF and PA in children compared to norm-referenced standards. ROC curves, as part of the examinee-centered standard setting approach, are used to determine the “optimal” or rather most appropriate cut-off point. Additionally, more than one cut-off point can be set in ROC analysis creating classification zones that enable a more valid evaluation of children with borderline test results. Because of the influence of body composition factors on PF and MC, BMI and Broca Index are reasonable health criteria for evaluating motor tests in children and thus for setting standards. Nevertheless, further research is still needed to confirm and verify a significant link between health factors and motor test scores and to avoid misinterpretation, longitudinal studies are required for this purpose.

ROC curves, based on the obesity risk factor and the respective motor test data, can deliver age- and sex-specific cut-off points for each FMC motor test item (see Section 3.3). As suggested by Welk et al. [30], the use of the sum of LMS-derived z-scores, in this case the BMI and the Broca Index, as health indicator in ROC analysis, may provide benefits by integrating normal growth and maturation into health-related CRS. After proving accuracy, e.g., by the area-under-the-curve, these cut-off points support the distinction of children at high or low risk of obesity. In addition to statistical calculations, pedagogical aspects need also to be taken into account in the standard setting process. Increased motivation and engagement for fitness training, movement skill development and an active lifestyle should be considered an important goal of motor testing in children. A holistic approach, that includes functional, process-, and product-oriented standards, supports to cover the multifaceted phenomenon of children’s gender- and age-specific physical abilities and motor skills. Finally, educational curricula that integrate health-related criterion-referenced motor testing, ensuring a high level of understanding of both the tests and its test standards among all stakeholders, as well as increased thinking outside the box, for example in terms of collaborations, will further enhance the value of PF and MC monitoring for children’s health. 

## 5. Limitations

The findings of this review have some limitations. A key limitation arises from the lack of data on health-related CRS for motor tests. Only five studies could be detected that set health-related cut-off points for the test items resulting in a low level of research fidelity that does not allow for significant interpretations. For the test items of stand-and-reach, 20 m-sprint, ball throwing, jumping sideways, balancing backwards and star run, no study could be found that set cut-off points based on health measures. Inversely, this certainly underlines the high relevance and urgency of standardized CRS for health-related motor tests. With the ROC curve method, it must be taken into account that cut-off points determined using ROC curves are only an approximation and that the unstable shape of ROC curves can lead to potentially misleading interpretations of the determined “optimal” cut-off point [76,77,84]. Moreover, differences in evaluation and scoring structures between different tests often limit the comparability of tests and their standards ((pp. 129–153 [32]), [41,42]). This again shows the high relevance of a globally standardized test and evaluation system.

Further, it needs to be kept in mind that there are multiple influence factors on physical abilities and motor skills. Naughton et al. [101] summarized factors that likely influence test results and include growth, test familiarization, cultural sensitivity, genetics, movement efficiency, nutrition, and cognitive age, among others. Consequently, these influence factors should not be neglected in the interpretation of test results and hence also of test standards.

## 6. Conclusions

In motor testing, high relevance is attributed to CRS in order to enable health-related evaluation, interpretation and subsequent valuable recommendations. In response to the lack of research and limited data on cut-off points for motor tests in children (see Section 3.1), implications for a methodological approach to establish health-related CRS for tests used in the Fulda Movement Check, but also applicable to other field-based tests, were developed in this article. 

ROC curves have recently been used to define cut-off points and health-related CRS. BMI-classification as well as the Broca Index are known as biomarkers for body composition in the field of obesity prevention and PF promotion. They represent reasonable health factors in criterion-referenced evaluation accordingly. Nevertheless, the correlation between the motor test and the health outcome must be subject to constant review. Furthermore, it is essential to consider pedagogical factors in the standard setting process to cover special characteristics of children in contrast to adults. Since motor tests are mostly conducted in school settings, the potential of demotivation, misunderstanding of outcomes and the gender-specific information processing of motor tasks must be kept in mind. 

Overall, assuming standardized test batteries, the standardized approach to setting health-related CRS complemented with further research in this field will form a fundamental base for a globally applicable evaluation and interpretation of health-related motor and fitness tests standards. Thereby, further increase in obesity can be counteracted and health care systems are supported in developing effective intervention and prevention programs. Additionally, education and enhanced knowledge about the relationship between PF, MC, PA, and obesity based on health-related criterion-referenced motor testing in children will help to promote the prevention of obesity and related health risks in further course of life.

## Figures and Tables

**Table 1:** Health-related cut-off points of Fulda Movement Check tests for children aged 7–10 years.

Motor Test Item	Author (Year)	Method	Health Indicator	Sample Size	Age	CRS Boys	CRS Girls
Sit-ups	The Cooper Institute (2017) [54] → Curl-Ups	Contrasting-groups method (trained vs. untrained)	Training response	*n* = N/A		[no. completed]	
					7 years. 8 years. 9 years. 10 years.	≥4 ≥6 ≥9 ≥12	≥4 ≥6 ≥9 ≥12
Standing long jump	Castro-Piñero et al. (2019) [55]	ROC	CVD risk score	*n* = 237	6–10 years.	≥104.5 cm	≥81.5 cm
6-min run	Santos Silva et al. (2020) [61] → 9-min run	ROC	BMI	*n* = 61.465		[9-min. (approximated 6 min.)]	
					6–8 years. 9–11 years.	≥1200 (800) m ≥1300 (867) m	≥1070 (713) m ≥1160 (773) m
Handgrip strength	Castro-Piñero et al. (2019) [55]	ROC	CVD risk score	*n* = 237	6–10 years.	≥0.367 kg/mass kg	≥0.306 kg/mass kg
	Fredriksen et al. (2018) [58]	ROC	CM risk	*n* = 2272	6–12 years.	handgrip strength = unsuitable predictor for cardiometabolic risk	
	Saint-Maurice et al. (2018) [59]	ROC	Bone health (TBLH_BMC)	*n* = 433 (USA) + 355 (ESP)	6 years. 7 years. 8 years. 9 years. 10 years.	>10.0 kg >11.2 kg >13.1 kg >15.2 kg >17.3 kg	>8.9 kg >10.2 kg >12.0 kg >14.0 kg >16.5 kg
Push-ups	The Cooper Institute (2017) [54]	Contrasting-groups method (trained vs. untrained)	Training response	*n* = N/A		[no. completed]	
					7 years. 8 years. 9 years. 10 years.	≥4 ≥5 ≥6 ≥7	≥4 ≥5 ≥6 ≥7

Legend: CRS = criterion-referenced standards; CVD = cardiovascular disease; CM = cardiometabolic; BMI = body mass index; TBLH_BMC = total body less head bone mineral content; ROC = receiver-operating-characteristic; N/A = not available.

**Table 2:** Methodological aspects of criterion-referenced standards in health-related motor testing.

Methodological Context	Author (Year)	Motor Test	Content/Theme
Body composition in motor testing	Zaqout et al. (2016) [65]	20-m-shuttle-run, handgrip strength, standing long jump, flamingo test, back-saver sit-and-reach, 40-m-sprint	Influence of physical fitness on cardiometabolic risk factors (IDEFICS study)
	Truter et al. (2010) [69]	PACER, sit-and-reach, standing long jump, knee push-ups, sit-ups, wall-sitting, aeroplane lying	Relationships of physical fitness and body composition classification
	Ervin et al. (2014) [72]	Handgrip strength, plank, modified pull-up, knee extension	Muscle strength and body weight
	Ruzbarska (2020) [66]	Balancing backwards, one-legged hopping, jumping laterally, moving sideways	Gross motor coordination in relation to weight status
	Tokmakidis et al. (2006) [63]	Sit-and-reach, standing long jump, sit-ups, agility-shuttle-run, 20-m-shuttle-run	Fitness levels in relationship to overweight and obesity
	Lovecchio & Zago (2019) [71]	Sit-and-reach, standing long jump, shuttle run 5-m × 10, sit-ups, bent arm hang	Fitness differences according to BMI categories
	Silva et al. (2018) [70]	20-m-shuttle-run	Criterion-referenced cut-points for low cardiorespiratory fitness associated with obesity
	Humberto P.-B. et al. (2019) [68]	20-m-shuttle-run	Cardiorespiratory fitness cut-off points related to body composition parameters
	Aires et al. (2008) [67]	Push-ups, sit-ups, trunk-lift, sit-and-reach, 20-m-shuttle-run	Association of physical fitness and body mass index in youth
	Maury-Sintjago et al. (2019) [62]	6-min-walk	Association between body mass index and functional fitness
Approaches to setting criterion-referenced standards and cut-off points	Zhu et al. (2011) [26]	Fitnessgram, without reference to a specific test	Approaches for development of criterion-referenced standards
	Plowman (1992) [73]	Sit-and-reach, pull-ups, sit-ups	Criterion-referenced standards for neuromuscular physical fitness tests
	Van Nijlen & Janssen (2008) [46]	Without reference to a specific test	Contrasting groups and Angoff-method
	Berk (1976) [74]	Without reference to a specific test	Determination of optional cutting scores in criterion-referenced measurement
	Cureton & Warren (1990) [52]	Mile run/walk test	Procedures used in development of criterion-referenced standards
	Plowman (2006) [51]	Fitnessgram, without reference to a specific test	Fitnessgram criterion-referenced standards
	Bös et al. (2006) [18]	MoMo: Push-ups, jumping sideways, standing long jump, balancing backwards, stand-and-reach, reaction, sticking-pins, tracing-lines, ergometer	Norm-referenced evaluation as a baseline approach (MoMo)
	Oberger & Bös (2009) [32] (pp. 129–153)	MoMo (see above)	Scaling and evaluation strategies (MoMo)
	Oberger (2015) [34]	MoMo (see above)	Setting standards, evaluation strategies, interpretation possibilities (MoMo)
	Going & Williams (1989) [19]	Mile run/walk, sit-ups	Understanding fitness standards
	Zhu et al. (2013) [44]	Fitnessgram, without reference to a specific test	Setting performance standards and cut-off scores
	Niessner et al. (2020) [35]	MoMo (see above)	Representative percentile curves of physical fitness from childhood to adulthood (MoMo)
	Kloe et al. (2020) [36]	20-m-sprint, 6-min-run	Percentile curves for 20 m-sprint and 6-min run
	Oberger et al. (2010) [15]	MoMo (see above)	Motor skills as a health indicator in children
	Safrit et al. (1980) [43]	Without reference to a specific test	Issues in setting motor performance standards
	De Miguel-Etayo et al. (2014) [75]	20-m-shuttle-run, handgrip strength, standing long jump, flamingo test, back-saver sit-and-reach, 40-m-sprint	Physical fitness reference standards in European children (IDEFICS study)
ROC curves	Welk et al. (2011) [30]	Fitnessgram, without reference to a specific test	New criterion-referenced standards for Fitnessgram tests using ROC
	Unal (2017) [76]	Without reference to a specific test	Defining an optimal cut-off point value in ROC analysis
	Mandrekar (2011) [77]	Without reference to a specific test	ROC curve in diagnostic test assessment
	Lang et al. (2019) [78]	20-m-shuttle-run	Criterion-referenced standards for cardiorespiratory fitness using ROC

## Data Availability

The data associated with the study are not publicly available but are available from the corresponding author on reasonable request.

## Appendix A

The full search strategy in Pubmed was designed based on the following search term modifiers: (“motor skill *” OR “motor coordination” OR “motor development” OR “motor performance” OR “motor activit *” OR “motor control” OR “motor proficienc *” OR “motor competenc *” OR “motor abilit *” OR “motor fitness” OR “motor function” OR “movement skill *” OR “gross motor *” OR “locomotor skill *” OR “fundamental motor skill *” OR “fundamental movement skill *” OR “physical fitness” OR “fitness” OR “physical performance” OR (“motor skill *”[MeSH Terms] OR “motor activit *”[MeSH Terms] OR “psychomotor performance”[MeSH Terms] OR “physical fitness”[MeSH Terms] OR “Physical Functional Performance”[MeSH Terms])) AND (“test batter” OR “Screen *” OR “health screen *” OR “health check” OR “Physical Health Assessment” OR “motor skill test” OR “performance diagnos *” OR “test” OR “assessment” OR “evaluat *” OR (“task performance and analysis”[MeSH Terms] OR “mass screening”[MeSH Terms] OR “diagnostic screening programs”[MeSH Terms])) AND (“health standard *” OR “reference value *” OR “cutoff value *” OR “cut-off value *” OR “cutoff scor *” OR “cut-off scor *” OR “cut-off point *” OR “cutoff point *” OR “cut point *” OR “criterion-referenced” OR “criterion-based” OR “criterion-oriented” OR “minimum threshold” OR “threshold value” OR “threshold” OR “minimal threshold” OR (“reference standard *”[MeSH Terms] OR “Differential Threshold”[MeSH Terms])) AND (“primary school” OR “elementary school” OR “child *” OR “student *” OR “kid” OR “youth” OR (“child”[MeSH Terms])).
